# Ultrafast Room Temperature Synthesis of Porous Polythiophene via Atmospheric Pressure Plasma Polymerization Technique and Its Application to NO_2_ Gas Sensors

**DOI:** 10.3390/polym13111783

**Published:** 2021-05-28

**Authors:** Choon-Sang Park, Do Yeob Kim, Eun Young Jung, Hyo Jun Jang, Gyu Tae Bae, Jae Young Kim, Bhum Jae Shin, Hyung-Kun Lee, Heung-Sik Tae

**Affiliations:** 1 Department of Electronics and Computer Engineering, College of Engineering, Kansas State University, Manhattan, NY 66506, USA; purplepcs@ksu.edu; 2 ICT Creative Research Laboratory, Electronics & Telecommunications Research Institute, Daejeon 34129, Korea; nanodykim@etri.re.kr; 3 School of Electronic and Electrical Engineering, College of IT Engineering, Kyungpook National University, Daegu 41566, Korea; eyjung@knu.ac.kr (E.Y.J.); bs00201@knu.ac.kr (H.J.J.); doctor047@knu.ac.kr (G.T.B.); jyk@knu.ac.kr (J.Y.K.); 4 Department of Electronics Engineering, Sejong University, Seoul 05006, Korea; hahusbj@sejong.ac.kr; 5 School of Electronics Engineering, College of IT Engineering, Kyungpook National University, Daegu 41566, Korea

**Keywords:** atmospheric pressure plasma, room temperature growth, plasma polymerization, porous polythiophene, conducting polymer, NO_2_, gas sensors

## Abstract

New nanostructured conducting porous polythiophene (PTh) films are directly deposited on substrates at room temperature (RT) by novel atmospheric pressure plasma jets (APPJs) polymerization technique. The proposed plasma polymerization synthesis technique can grow the PTh films with a very fast deposition rate of about 7.0 μm·min^−1^ by improving the sufficient nucleation and fragment of the thiophene monomer. This study also compares pure and iodine (I_2_)-doped PTh films to demonstrate the effects of I_2_ doping. To check the feasibility as a sensing material, NO_2_-sensing properties of the I_2_-doped PTh films-based gas sensors are also investigated. As a result, the proposed APPJs device can produce the high density, porous and ultra-fast polymer films, and polymers-based gas sensors have high sensitivity to NO_2_ at RT. Our approach enabled a series of processes from synthesis of sensing materials to fabrication of gas sensors to be carried out simultaneously.

## 1. Introduction

Recently, conducting polymers, such as polyaniline (PAni), polypyrrole (PPy), polythiophene (PTh), and their derivatives, have attracted attention to researchers as sensing materials of toxic gases [1,2,3,4]. In comparison with most of the commercial gas sensors, which are usually based on metal oxides and operated at high temperatures, the gas sensors made of conducting porous polymers have many advantages [5,6,7]. The advantages of conducting polymers compared to inorganic materials used until now are their diversity, their easy synthesis, and particularly, their sensitivity at room temperature. Conducting polymers can be synthesized by various techniques, such as chemical synthesis, electrochemical method, hard and soft templates, interfacial polymerization, and plasma polymerization [1]; however, some of conducting polymers are interactable and soluble in few kinds of solution [8]. Therefore, in-situ polymerization of conducting polymers on substrates with nanostructures, such as pore network or perpendicular pillar structure without external heat or association of other processes, is important for the preparation of gas sensor devices. Very recently, we have developed a nano-porous polymer synthesis method with dry process using a novel atmospheric pressure plasma jets (APPJs) with additional guide tube and bluff body [9,10,11,12,13,14,15,16]. The nitrogen-containing polymer nanoparticles were successfully synthesized by using aniline and pyrrole monomers via an APPJs polymerization technique. The plasma polymerizations of aniline and pyrrole have been successfully implemented using intense and broad glow-like plasma [10,11,12,13,14,15]. However, there is no report on the synthesis of sulfur (S)-containing conjugated PTh through the novel APPJs polymerization technique. In addition, to the authors‘ knowledge, there have been no previous reports on the investigation of the gas sensors based on conducting nano-porous polymers prepared by APPJs polymerization.

Accordingly, this study uses field emission-scanning electron microscopy (FE-SEM), atomic force microscopy (AFM), Fourier transform-infrared spectroscopy (FT-IR), X-ray photoelectron spectroscopy (XPS), and time of flight-secondary ion mass spectrometry (ToF-SIMS) to analyze pure and iodine (I_2_)-doped PTh films synthesized by APPJ polymerization. We also investigate the NO_2_-sensing properties of the I_2_-doped conducting porous PTh films in order to check the feasibility for sensing materials of gas sensors.

## 2. Experimental

### 2.1. Atmospheric Pressure Plasma Jets (APPJs) for Synthesis of Polythiophene

In the previous work, in order to produce intense glow-like plasma by using novel APPJs, we use a guide tube and polytetrafluoroethylene bluff body installed at the jet end to confine the jet flow and to minimize the quenching from ambient air. Consequently, the novel APPJs can expand farther downstream in nucleation region and can produce the broad and intense glow-like plasma during plasma polymerization (Figure S1 in the Supplementary Material). In the case of APPJs without the proposed guide and bluff body, the plasma was only produced within the area of the array jets due to the directional properties of the streamer-like plasma discharges. Whereas, in the case of the APPJs with proposed guide tube and bluff body (impinging jet) systems, the intense and broaden plasma was produced within the whole area of the impinging region, thereby increasing the plasma region about 60-fold and the deposition area. This APPJs with impinging technique can produce a broadened and intense plasma discharge with large area and homogeneous deposition of polymers. The detailed plasma polymerization method using a stationary APPJs with a guide tube and bluff body was previously described in detail [9,10,11,12,13,14,15]. During polymerization experiments by using APPJs, the plasma jet was not moved, and as such, experiments were conducted in a stationary deposition. Argon gas was employed as the discharge gas for plasma generation and its flow rate was fixed to 1700 standard cm^3^/min (sccm). Liquid thiophene monomer was vaporized by means of a glass bubbler, which was supplied by argon gas with the flow rate of 170 standard cm^3^/min. When the mass flow rate of the monomer was applied higher than the optimal condition (170 standard cm^3^/min), the discharge was disturbed and becomes unstable even though the proposed guide tube and bluff body with impinging jet systems was used. Whereas, when the mass flow rate of the monomer was applied lower than the optimal condition, the deposition rate was decreased. The PTh films were obtained at a sinusoidal wave with a peak value of 8 kV and a frequency of 26 kHz under ambient air. The pure PTh films using APPJs without any proton donor doping has been an insulating state. Therefore, it is necessary to introduce charge carriers into the plasma polymerized structures to render them conductive by using I_2_ doping method and require further examination for the formation of novel pure and I_2_-doped PTh materials using a novel APPJs technique. For I_2_ doping, PTh samples were placed in a sealed glass container containing 2 g of solid I_2_ crystals for 30 sec at room temperature immediately after plasma polymerization.

### 2.2. Instruments

The field emission-scanning electron microscopy (FE-SEM; Hitachi SU8220, Hitachi, Tokyo, Japan) was employed to analyze the planar and cross-sectional morphologies of the synthesized PTh films with an accelerating voltage and current of 5 kV and 10 mA, respectively. A conductive platinum coating was used when imaging the samples. The photographs of the device and plasmas were taken using a digital camera (D5300, Nikon, Tokyo, Japan) with a Macro 1:1 lens (SP AF 90 mm F2.8 Di, Tamron, Saitama, Japan). AFM (NX20, Park Systems) was used to monitor the surface roughness based on three-dimensional (3D) pure and I_2_-doped PTh surface images. The scan rate was set at 0.5 Hz and the scanning area was 20 μm × 20 μm. AFM data was analyzed using the i-solution software (IMT i-solution Inc., Burnaby, BC, Canada) to obtain the distribution of granular sizes (granularity cumulation) in the deposited PTh film. The molecular structure of the PTh film on the glass was taken by using a Fourier transformation infrared spectroscopy (FT-IR; Vertex 70, Bruker, Berlin, Germany) at the Korea Basic Science Institute (KBSI; Daegu, Korea). FT-IR spectra were measured by averaging 128 scans at a wavenumber resolution of 0.6 cm^−1^ using attenuated total reflection (ATR) mode between 500 and 4000 cm^−1^ to determine the chemical difference in the pure and I_2_-doped PTh films. The XPS was carried out on an ESCALAB 250XI surface analysis system (Thermo Fisher Scientific, Waltham, MA, USA), using monochromatic Al Kα X-ray source (hυ = 1486.71 eV) operated at 15 kV and 20 mA. The pressure in the analyzing chamber was maintained at 10^−7^ Pa or lower during analysis and the size of the analyzed area was 500 μm × 500 μm. Spectra were acquired with the angle between the direction of the emitted photoelectrons and the surface equal to 60°. The estimated analyzing depth of the used XPS set up was 8 to 10 nm. The high-resolution spectra were taken in the constant analyzer energy mode with a 200 eV for survey scan and a 50 eV pass energy for element scan, respectively. The value of 285.8 eV of the C 1s core level was used for calibration of the energy scale. To curve fit the high-resolution C 1s, S 2p, and O 1s peaks, the deconvolution of C 1s, S 2p, and O 1s peaks was analyzed by the Thermo Advantage software. The peaks were deconvoluted using Gaussian–Lorentzian peak shapes (constrained between 80% and 100% Gaussian) and the full-width at half maximum (FWHM) of each line shape was constrained between 2.0 and 3.0 eV. The ToF-SIMS data were obtained using a ToF-SIMS V instrument (ION-TOF GmbH, Münster, Germany) equipped with a reflectron analyzer, a bismuth primary-ion (Bi_3_^+^) source and a pulsed electron flood source for charge compensation. The pressure in the analysis chamber was maintained at less than 7.5 × 10^−12^ Pa. Bi_3_^+^ (0.5 pA) accelerated at 30 keV was used as the analysis (primary) gun. Negative-ion and positive-ion mass spectra were acquired from a 500 μm × 500 μm area using a Bi_3_^+^ primary-ion beam operating at 30 keV. The mass resolution was typically greater than 8000 at *m/z* = 29 Si. Secondary ions were detected in negative ion mode and a full spectrum from 1 to 2000 amu was acquired.

### 2.3. Sensor Fabrication and Measurement of Gas-Sensing Properties

The alumina substrates supplied with Pt interdigitated electrodes (IDEs) were ultrasonically cleaned in acetone and deionized water for 10 min, respectively. Au wires were bonded to two contact pads of the IDEs using an Au conducting paste and then heating at 500 °C for 30 min was conducted to enhance the adhesion between the Au wires and contact pads of the IDEs. The PTh films as a sensing material were directly deposited onto the alumina/IDEs substrates using APPJs polymerization. The fabricated sensor devices were located in a gas measurement chamber without any external heater. Air with 65% relative humidity was used as a balance gas at a flow rate of 1000 sccm, while 50 ppm of NO_2_ gas was used as an analyte. The analyte was diluted with the balance gas to achieve the desired concentration of 0.25 to 2 ppm using mass flow controllers. The resistance of the PTh based gas sensors was recorded using a data acquisition system consisting of Agilent 34970A and BenchLink Data Logger software during the gas measurement.

## 3. Results and Discussion

**Figure 1:**
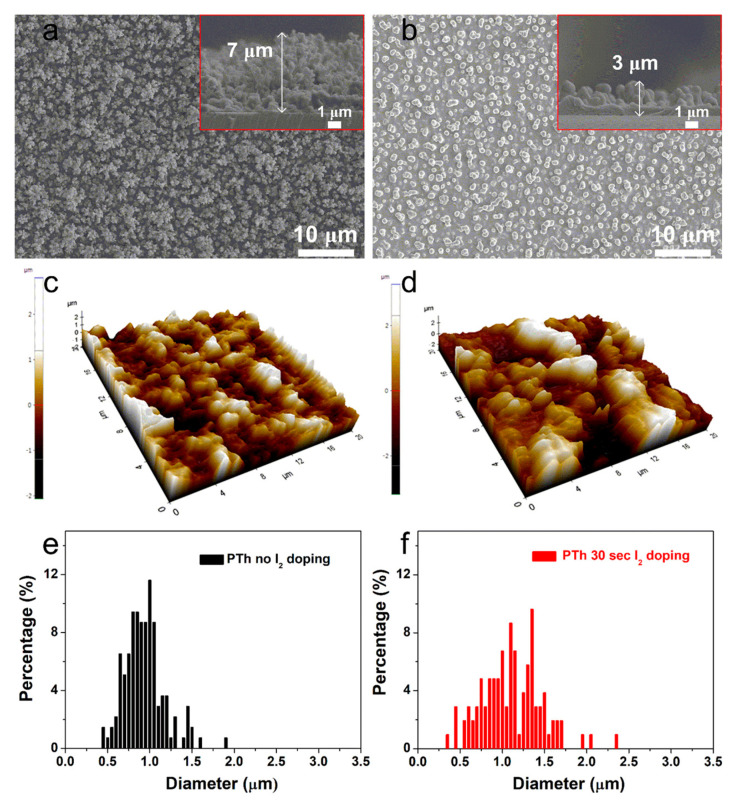
Planar and cross-sectional (insets) SEM images of the plasma polymerized thiophene (PTh) films (**a**) without and (**b**) with I_2_ doping. AFM images of the PTh films (**c**) without and (**d**) with I_2_ doping. Granularity cumulation distribution charts obtained from AFM images of the PTh films (**e**) without and (**f**) with I_2_ doping.

Figure 1a shows the planar and cross-sectional SEM images of the PTh films deposited at RT for 60 s through novel APPJs polymerization technique. The pure (undoped) PTh films consisted of dense nanoparticles with porous networks and showed very fast deposition rates of about 7 μm·min^−1^ (inset of Figure 1a). Most of nanoparticles vanished after I_2_ doping for 30 sec as shown in Figure 1b and the thickness of the PTh films decreased to 3 μm (inset of Figure 1b). To further investigate the characteristics of the PTh films, roughness (R_rms_) and grain diameter of PTh films were studied by the AFM measurement as can be seen in Figure 1c–f. The pure PTh surface showed relatively small-size protrusions and the surface R_rms_ was 370 nm. On the other hand, after I_2_ doping for 30 sec, lots of large protrusions appeared on the PTh surface and the R_rms_ increased to 890 nm. In addition, as shown in the granularity cumulation distribution charts in Figure 1e,f, the diameter range for the pure PTh sample was between 0.4 and 1.8 μm. After I_2_ doping, however, the diameter range became wider, between 0.3 and 2.3 μm due to particle aggregation, thereby increasing the grain size. The increase in the average grain diameter from 0.8 to 1.1 μm after I_2_ doping means that I_2_ doping decreases the number of grain boundaries. This decreased number of grain boundaries was mainly due to particle aggregation and disconnected networks between adjacent nanofibers from higher porosity. For this iodine doping process, the particles were aggregated due to the hydration and I_2_ particles. The surface morphology of the doped PTh film was changed a more globular particle and rougher surface by an incorporation of iodine. The surface of the doped PTh film presents large grains separated by smooth areas. This type of topography can be attributed to the branched molecular geometry of the doped PTh film, which would facilitate the formation of granular agglomerates. These results are similar to the tendency of the APP polymerized polyaniline nanoparticles vanished and aggregated after I_2_ doping [10]. Thereby, the surface morphology of the doped PTh film was changed into a more globular particle owing to the aggregated particles by an incorporation of iodine [10,17,18].

**Figure 2:**
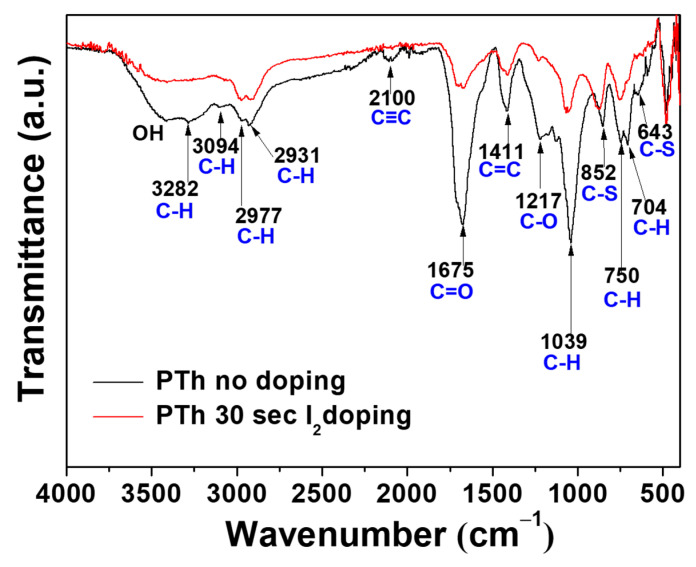
FT-IR spectra of the plasma polymerized thiophene films without and with I_2_ doping.

In order to identify chemical composition of PTh films, we analyzed the films by using FT-IR and XPS techniques. Figure 2 shows the obtained FT-IR spectra of the PTh films synthesized by the novel APPJs polymerization technique. The small peak of the C–H stretching vibration of the alkyne bond was observed in the peak at 3282 cm^−1^. Furthermore, the peak at about 2100 cm^−1^, which in that case could be attributed to triple bond C≡C stretching of alkyne functions, seems to be present in pure PTh film [18,19,20]. The spectrum of polymer contains the peak at 3094 cm^−1^ that can be assigned to the C–H stretching of the aromatic proton bands within the thiophene ring. The two small peaks at 2977 and 2931 cm^−1^ can be also assigned to the C–H stretching vibration. A peak belonging to the C=O symmetric stretching vibration modes of thiophene ring was located at 1675 cm^−1^ [19]. This carbonyl group is formed due to the reaction of thiophene and oxygen in ambient air. This functional group provides the hydrophilic into the film. Therefore, water from the ambient air is absorbed, thus resulting in forming the O–H stretching bond at around 3500 cm^−1^ in the PTh film [19,20]. The peak at 1411 cm^−1^ is a characteristic of the aromatic C=C stretching vibration. A peak belonging to the C–O stretching bond was located at 1217 cm^−1^. The peaks at 1039, 750, and 704 cm^−1^ are related to in-plane and out-of-plane C–H deformation vibration in the thiophene ring. The two peaks at 852 and 643 cm^−1^ can be assigned to the C–S bending [21,22,23,24,25]. Those results confirm the presence of thiophene ring in the PTh film structure. The absorption peaks at 1675 and 1217 cm^−1^ confirm the inclusion of small amounts of oxygen in the films, incorporated during plasma deposition [25]. These small amounts of oxygen could have originated in the oxidation of the PTh from ambient air [19,20]. After I_2_ doping for 30 sec, most of the spectra peaks significantly diminished and became smooth, presumably due to the absorption of hydrogen in the PTh films via the I_2_ doping. Iodine probably reacted with residual radicals in the PTh films by the doping. Iodine radicals can also be formed by the hemolytic dissociation of iodine. As iodine radicals can extract hydrogen atoms from the PTh structure, this could change the bonding characteristics of plasma polymerized films [18,20,21,22,23,24,25,26,27]. In particular, hydrogen iodide formed by reaction of iodine radicals and hydrogen atom can react with C≡C bond, thus causing the disappearance of C≡C bond after I_2_ doping [20]. In addition, after I_2_ doping process, the decrease of spectra intensities is presumably due to the decrease in the relative amounts of surface reflection, which is originated by film properties, such as particle size and surface roughness of the film [28].

**Figure 3:**
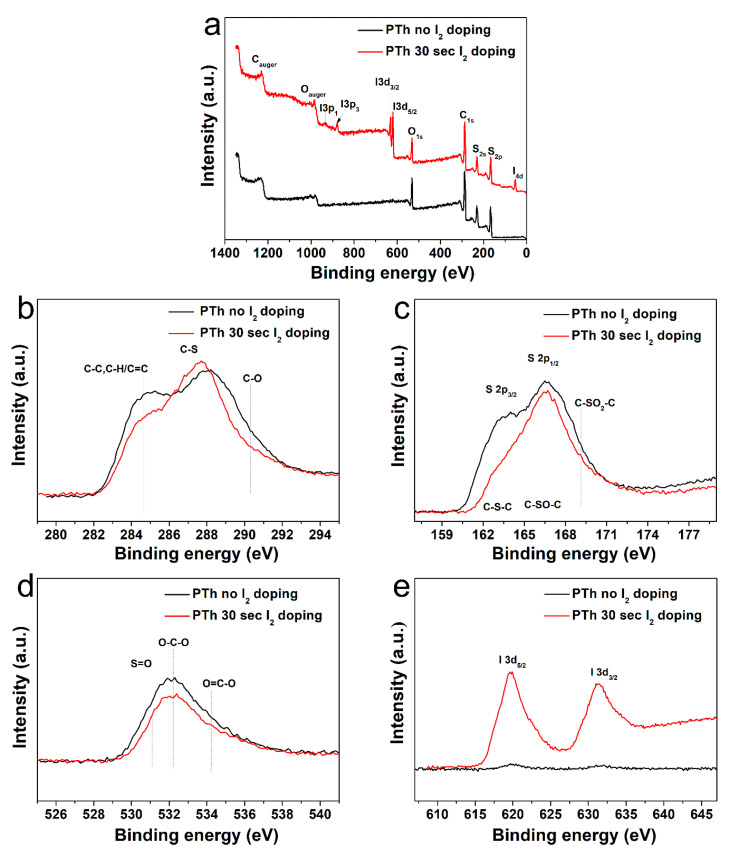
(**a**) XPS spectra of the plasma polymerized thiophene films without and with I_2_ doping. High-resolution XPS spectra of (**b**) C 1s, (**c**) S 2p, (**d**) O 1s, and (**e**) I 3d.

Chemical composition of the PTh films obtained by FT-IR spectra was analyzed by comparing the XPS results. In Table 1 and Figure 3a, the elemental concentration in atomic percentages and survey spectra identified from XPS are presented. The high-resolution C 1s, S 2p, O 1s, and I 3d peaks were analyzed in detail, as presented in Figure 3b–e and Figure S2 in the Supplementary Material. The peak assignments and envelope compositions of various C 1s, S 2p, and O 1s were summarized in Table 2, Table 3 and Table 4, respectively. The XPS survey spectra in Figure 3a show signals corresponding to C 1s (285.5 eV), S 2s (228.0 eV), S 2p (164.0 eV and 168.5 eV), O 1s (532.1 eV), I 3d_3/2_ (620.6 eV), I 3d_5/2_ (631.0 eV), and I 4d (53.0 eV) electronic orbitals. The presence of O atoms in the PTh plasma polymerized films is expected because we synthesized the PTh films under ambient air and the films obtained through APPJs polymerization technique are an oxidized state of polymer, which are in agreement with the FT-IR data. The C 1s peak could be divided into three distinctive component peaks (Figure 3b and Figure S2 in the Supplementary Material, and Table 2) attributed to C–C, C=C, C–H bonds (284.7 eV), C–S bond (287.5 eV), and C–O bond (290 eV) [25]. The S 2p peak could be split into three component peaks (Figure 3c and Figure S2 in the Supplementary Material, and Table 3) mainly contributed by aromatic sulfide (C–S–C, 163.6 eV), sulfoxide (C–SO–C, 166.38 eV), and sulfone (C–SO_2_–C, 168.9 eV) [29]. The O 1s peak was decomposed in three peaks (Figure 3e and Figure S2 in the Supplementary Material, and Table 4). From the spectra of O 1s, it could be seen that S-doped carbon displays the same oxygen species of O–C–O (532.3 eV) and O=C–O (534.7 eV). In addition, it also had a characteristic peak at 531.1 eV, which was attributed to the S-containing group S=O [29,30]. The XPS spectra exhibited by I 3d core of the I_2_-doped PTh are shown in Figure 3e, which shows the location of I 3d_5/2_ and I 3d_3/2_ peaks at the binding energy of 620.6 and 631.0 eV, respectively. The location of these peaks and the peak separation of 10.4 eV strongly suggest the I_2_-doped polymer characteristics [30,31]. After I_2_ doping for 30 sec, the carbons with oxidation, such as C–O and O–C–O, were remarkably decreased, which showed more hydrophobic characteristics [10,15]. The atomic percentage of C–C, C–H, and C=C bonds decreased from 39.9 to 31.7%, indicating some disruption to the conjugated framework of the films [31]. Whereas, S-containing groups with carbon and oxygen, such as C–S, C–SO–C, C–SO_2_–C, and S=O except for C–S–C, increased when adopting the I_2_ doping. Therefore, total amounts of oxygen decrease only slightly. In addition, since iodine could easily absorb hydrogen from materials, this reduced the C–H bond.

**Figure 4:**
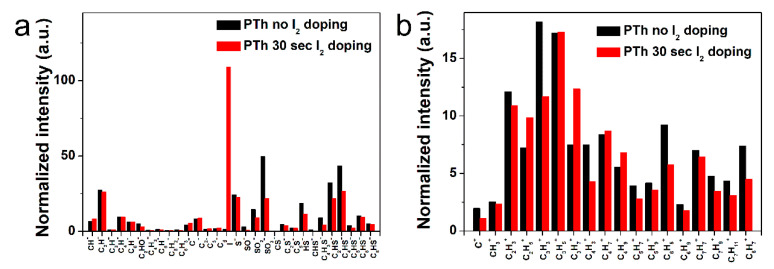
Normalized intensities of (**a**) negative-ion spectra and (**b**) positive-ion spectra of ToF-SIMS on surface of the plasma polymerized thiophene films without and with I_2_ doping.

In order to determine the specific chemical structures between the pure and I_2_-doped PTh films after the APPJs polymerization, the PTh films were characterized in both the positive and negative ion modes using ToF-SIMS. In Figure 4 and Figure S3 in the Supplementary Material, the negatively and positively charged ions static mass spectra and normalized intensities of ToF-SIMS for the PTh films are presented. The assignments of the selected peaks of PTh detected in the negative and positive mode are shown in Tables S1 and S2 in the Supplementary Material. For the PTh features, several characteristic peaks from the polymer chain were detected. In Figure S3a,b in the Supplementary Material, many ion peaks of the S-containing group were detected at *m/z* = 32, 33, 44, 45, 48, 56, 57, 64, 80, 81, 83, 92, 93, 105, and 129 amu and attributed to S^−^, HS^−^, CS^−^, CHS^−^, SO^−^, C_2_S^−^, C_2_HS^−^, SO_2_^−^, C_4_HS^−^, C_4_H_3_S^−^, C_5_S^−^, C_5_HS^−^, C_6_HS^−^, and C_8_HS^−^, respectively, which were characteristics of PTh fragments. The negative ions were related with abundant secondary ions C_2n_HS^−^ (n = 0–4) which were the fragments of PTh backbones (with alkyl side chains). The C_2n_HS^−^ ions show that polythiophene backbones were easily accessed by ToF-SIMS. In addition, the secondary ion fragments C_2n_H^−^ (*n* = 0–4) of alkyl side chains (and S- from thiophene ring) were detected (Figure 4a) [15,32]. Figure S3c,d in the Supplementary Material show the series of hydrocarbon fragments arising from PTh, such as CH_3_^+^, C_2_H_3_^+^, C_2_H_5_^+^, C_3_H_3_^+^, C_3_H_5_^+^, C_3_H_7_^+^, C_4_H_3_^+^, C_4_H_5_^+^, C_4_H_7_^+^, C_4_H_9_^+^, C_5_H_7_^+^, C_5_H_9_^+^, C_6_H_5_^+^, C_6_H_9_^+^, C_7_H_7_^+^, C_7_H_9_^+^, and C_7_H_11_^+^. These clusters are typical aliphatic hydrocarbon fragments of the form C_n_H_2n−3_, C_n_H_2n−1_, and C_n_H_2n+1_, arising from the PTh chain (Figure 4b). After I_2_ doping for 30 sec, almost all normalized intensities were decreased, presumably due to the absorption of hydrogen in the PTh films via the I_2_ doping. In addition, few negative and positive ion peaks of the oxygen-containing group were detected, which was likely to originate from the oxidation of particles from atmospheric pressure during the APPJs polymerization. These results agreed well with the FT-IR and XPS analyses.

To check the feasibility as a sensing material for the detection of poisonous gas, we investigated NO_2_ sensing characteristics of the I_2_-doped PTh films at RT. The gas response is defined as follows. Response (%) = (R_a_ − R_g_)/R_a_ × 100, where R_a_ and R_g_ are resistances of the PTh films before and after exposure to NO_2_ gas, respectively.

**Figure 5:**
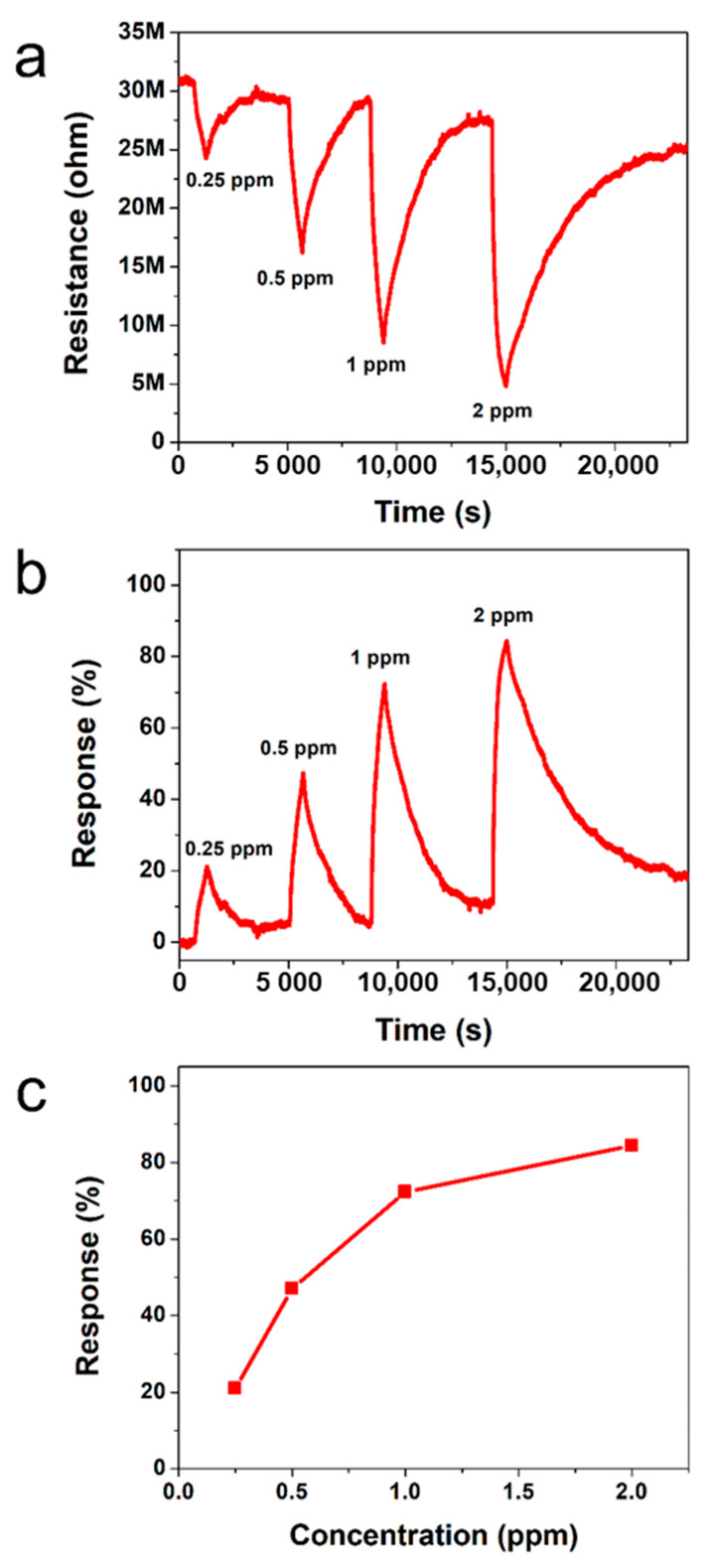
Transient (**a**) resistance and (**b**) response of sensors based on I_2_-doped PTh films to different NO_2_ concentrations at RT. (**c**) Responses as a function of NO_2_ concentration.

Figure 5a,b show the dynamic resistance and response of the gas sensors to various NO_2_ concentrations ranging from 0.25 to 2 ppm, respectively. It is observed that the exposure of PTh films to NO_2_ results in a decrease in value of resistance and the resistance of the PTh films gradually recover to its initial value when NO_2_ gas is turned off. It is known that PTh is a *p*-type material and NO_2_ is an oxidizing gas (electron acceptor). When PTh interacts with NO_2_, the concentration of majority charge carriers (holes) increases, which implies that the conductivity of *p*-type PTh increases. The initial base resistance (~31 MΩ) shifts toward lower resistance (~28 MΩ right before exposure to 2 ppm) with an increase in the NO_2_ concentration because the longer time is required for full recovery. Figure 5c shows the response of PTh films as a function of NO_2_ concentration. The gas response is increased with an increase in the NO_2_ concentration and the response of the PTh films was 21, 47, 72, and 84% for the NO_2_ concentration of 0.25, 0.5, 1, and 2 ppm, respectively. The gas sensors reported here exhibit highly sensitive behavior to NO_2_ compared to PTh based sensors reported previously, as shown in Table 5 [33,34,35]. To examine the repeatability of the sensor based on I_2_-doped PTh films, the NO_2_-sensing measurement was repeated, as shown in Figure S4 in the Supplementary Material. The difference in response between the first and second measurements at each concentration was within 5%, indicating good repeatability of the sensor based on I_2_-doped PTh films.

We think that the high response of PTh through APPJs polymerization comes from porous structure of PTh resulting from in-situ polymerization on the substrate. Such a higly porous structure assists NO_2_ in interacting with the surface of PTh films as well as the inside of porous network. As a result, new nanostructured conducting porous polymer sensor prepared using novel APPJs polymerization technique showed the excellent NO_2_-sensing properties at RT. In addition, our approach enables a series of processes from synthesis of sensing materials to fabrication of gas sensors to be carried out simultaneously.

## 4. Conclusions

In this study, porous PTh films were successfully synthesized from liquid thiophene monomer by using novel APPJs polymerization technique and demonstrated as a sensing material for NO_2_ detection. The PTh films consisted of dense nanoparticles with porous networks and had ultra-high fast deposition rates of about 7.0 μm·min^−1^. Spectroscopic analyses (FT-IR, XPS, and ToF-SIMS) reflect the retention of the aromatic ring and fragments of backbones in the structure of PTh films. As a result, new nanostructured conducting porous PTh sensor prepared using novel APPJs polymerization technique with I_2_ doping showed the excellent NO_2_-sensing properties at RT.

## Figures and Tables

**Table 1:** Elemental concentration in atomic percentages of PTh films without and with I_2_ doping observed in XPS spectra in Figure 3a.

Sample	Elemental Concentration			
	C 1s (at.%)	S 2p (at.%)	O 1s (at.%)	I 3d (at.%)
PTh without I_2_ doping	67.8	20.9	11.3	0.0
PTh with I_2_ doping	68.4	18.6	11.0	2.0

**Table 2:** Peak assignment (BE, eV) and envelope composition (%, total = 100) of various C 1s core level spectra of PTh films observed in XPS in Figure 3b.

Sample	C 1s Peaks Assignment and Envelope Composition		
	284.70 C–C, C–H, C=C	287.53 C–S	290.03 C–O
PTh without I_2_ doping	39.9	43.5	16.6
PTh with I_2_ doping	31.7	56.9	11.4

**Table 3:** Peak assignment and envelope composition of various S 2p core level spectra of PTh films observed in XPS in Figure 3c.

Sample	S 2p Peaks Assignment and Envelope Composition		
	163.62 C–S–C	166.38 C–SO–C	168.99 C–SO_2_–C
PTh without I_2_ doping	39.7	46.8	13.5
PTh with I_2_ doping	27.1	57.0	15.9

**Table 4:** Peak assignment and envelope composition of various O 1s core level spectra of PTh films observed in XPS in Figure 3d.

Sample	O 1s Peaks Assignment and Envelope Composition		
	531.12 S=O	532.38 O–C–O	534.74 O=C–O
PTh without I_2_ doping	15.7	58.5	25.8
PTh with I_2_ doping	18.4	52.7	28.9

**Table 5:** NO_2_ responses of PTh based gas sensors.

Sensing Material	Concentration	Temperature	Response	Ref
PTh	1 ppm	RT	3%	1
PTh	10 ppm	RT	9%	2
PTh	10 ppm	RT	8%	3
Poly(3-hexylthiophene)	20 ppm	RT	10%	4
PTh	1 ppm	RT	72%	This work

## Data Availability

The data presented in this study are available on request from the corresponding author.

## Supplementary Materials

The following are available online at https://www.mdpi.com/article/10.3390/polym13111783/s1, Figure S1. A photograph of the plasma produced with supply of vaporized thiophene monomer, Figure S2. The detailed high-resolution with deconvolutions of C 1s, S 2p, and O 1s spectra in Figure 3 of the plasma polymerized thiophene films (a) without and (b) I_2_ doping, Figure S3. Negative-ion spectra (0-150 amu) and positive-ion spectra (0-150 amu) of ToF-SIMS on surface of the plasma polymerized thiophene films without and with I_2_ doping. (a) Negative-ion without I_2_ doping. (b) Negative-ion with I_2_ doping. (c) Positive-ion without I_2_ doping. (d) Positive-ion with I_2_ doping, Figure S4. Repeatability of the sensors based on I_2_-doped PTh films at different NO_2_ concentrations, Table S1. Selected peaks and their assignments observed in negative-ion time of flight secondary ion mass spectrometry (ToF-SIMS) spectra of the PTh films, Table S2. Selected peaks and their assignments observed in positive-ion ToF-SIMS spectra of the PTh films.
