# Supplementary material for: Ultrafast Room Temperature Synthesis of Porous Polythiophene via Atmospheric Pressure Plasma Polymerization Technique and Its Application to NO2 Gas Sensors
Source: Polymers (Basel). 2021 May 28;13(11):1783. doi: 10.3390/polym13111783 (PMC8197993; doi:10.3390/polym13111783)
Supplement: Supplementary file 1 [file polymers-13-01783-s001.zip › polymers-1224107-supplementary.pdf]

polymers

Supplementary Materials

Ultrafast Room Temperature Synthesis of Porous Polythiophene via Atmospheric Pressure Plasma Polymerization Technique and Its Application to NO_2 Gas Sensors

Choon-Sang Park [1,†], Do Yeob Kim [2,†], Eun Young Jung [3], Hyo Jun Jang [3], Gyu Tae Bae [3], Jae Young Kim [3],
Bhum Jae Shin [4], Hyung-Kun Lee [2,*] and Heung-Sik Tae [3,5,*]

1 Department of Electronics and Computer Engineering, College of Engineering, Kansas State University, Manhattan, NY 66506, USA; purplepcs@ksu.edu
2 ICT Creative Research Laboratory, Electronics & Telecommunications Research Institute, Daejeon 34129, Korea; nanodykim@etri.re.kr
3 School of Electronic and Electrical Engineering, College of IT Engineering, Kyungpook National University, Daegu 41566, Korea; eyjung@knu.ac.kr (E.Y.J.); ; bs00201@knu.ac.kr (H.J.J.); doctor047@knu.ac.kr (G.T.B.); jyk@knu.ac.kr (J.Y.K.)
4 Department of Electronics Engineering, Sejong University, Seoul 05006, Korea; hahusbj@sejong.ac.kr
5 School of Electronics Engineering, College of IT Engineering, Kyungpook National University, Daegu 41566, Korea
* Correspondence: hklee@etri.re.kr (H.-K.L.); hstae@ee.knu.ac.kr (H.-S.T.); Tel.: +82-53-950-6563 (H.-S.T.)
† These authors contributed equally to this work.

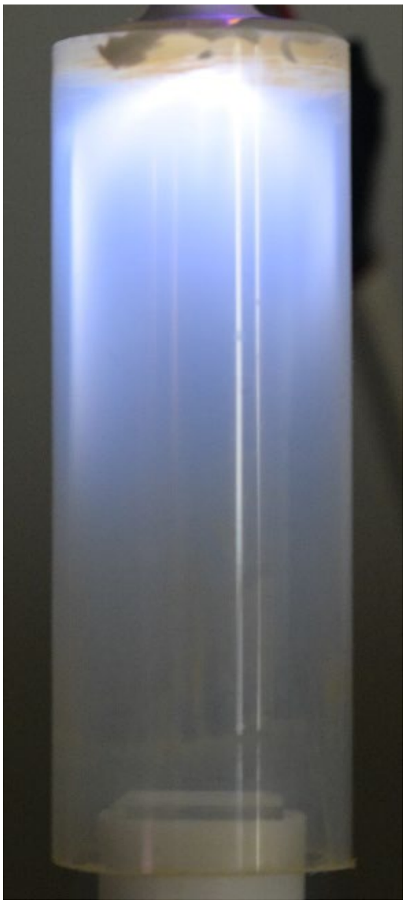

Figure S1. A photograph of the plasma produced with supply of vaporized thiophene monomer.

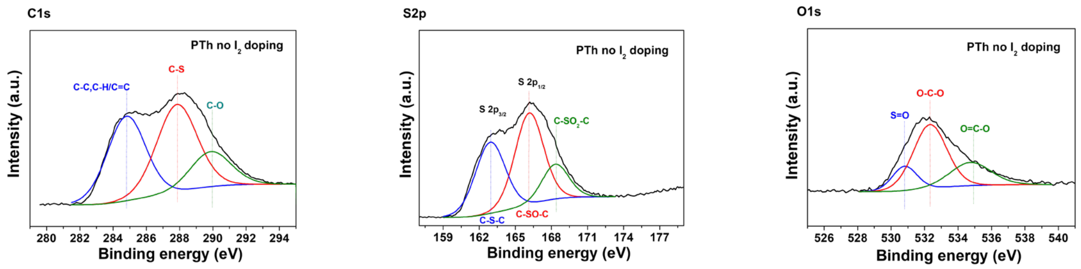

(a)

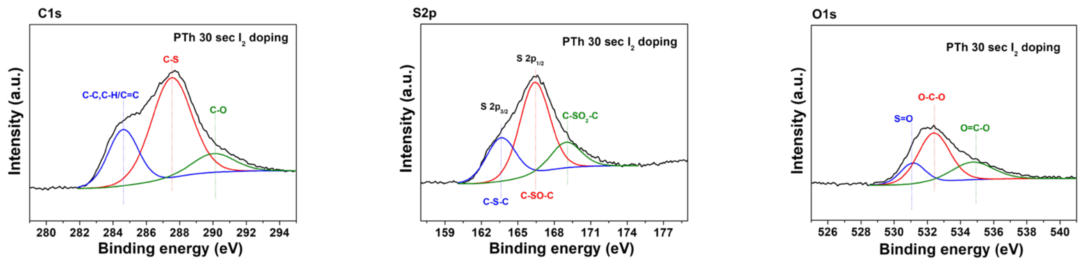

(b)

Figure S2. The detailed high-resolution with deconvolutions of C 1s, S 2p, and O 1s spectra in Figure 3 of the plasma polymerized thiophene films **(a)** without and **(b)** I_2 doping.

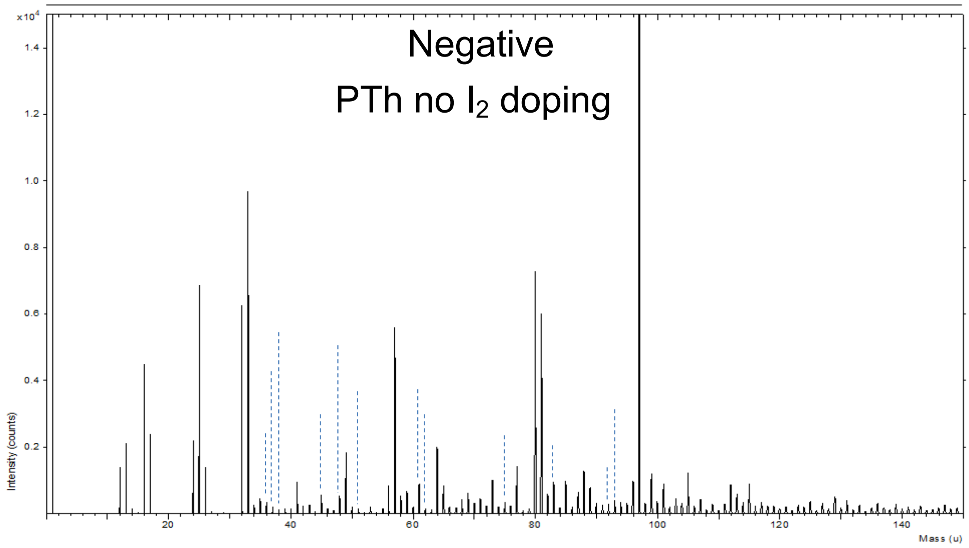

(a)

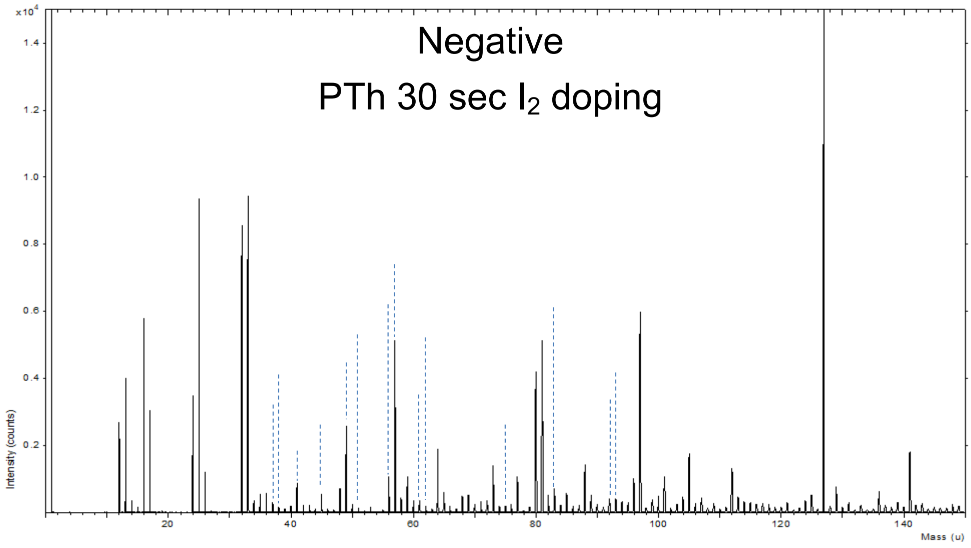
Negative
PTh 30 sec I2 doping
Intensity (counts)
Mass (u)

(b)

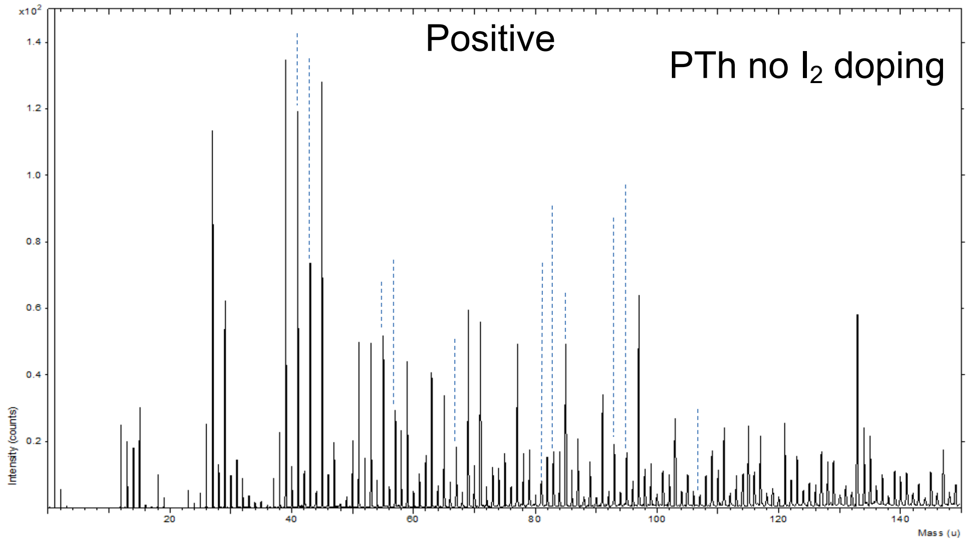
Positive
PTh no I2 doping
Intensity (counts)
Mass (u)

(c)

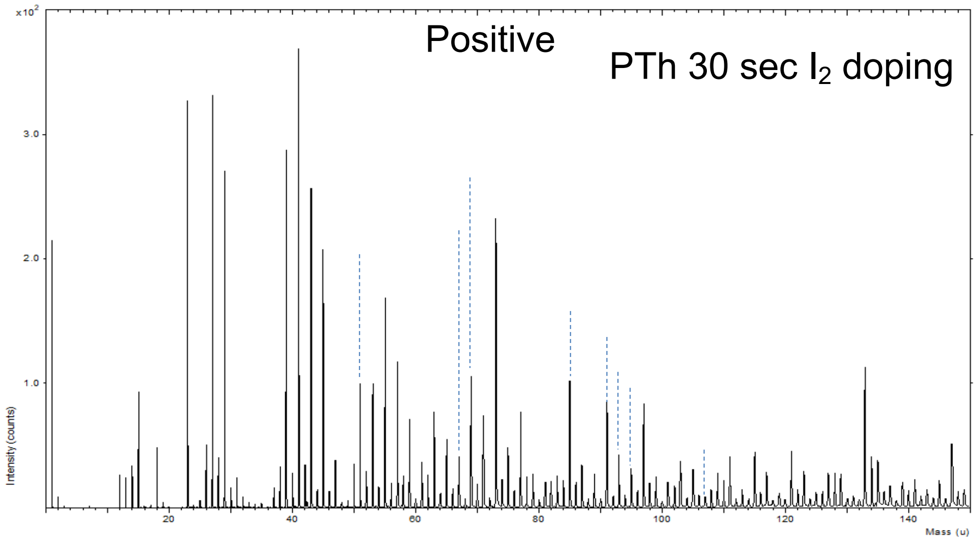
Positive
PTh 30 sec I2 doping
Intensity (counts)
Mass (u)

(d)

Figure S3. Negative-ion spectra (0-150 amu) and positive-ion spectra (0-150 amu) of ToF-SIMS on surface of the plasma polymerized thiophene films without and with I_2 doping. (**a**) Negative-ion without I_2 doping. (**b**) Negative-ion with I_2 doping. (**c**) Positive-ion without I_2 doping. (**d**) Positive-ion with I_2 doping.

Table S1. Selected peaks and their assignments observed in negative-ion time of flight secondary ion mass spectrometry (ToF-SIMS) spectra of the PTh films.

Negative ion mass spectrum *m*/*z*	Possible ion fragment/Possible structure
1	H^-
12	C^-
13	CH^-
15	NH^-
16	O^-
17	OH^-
24	C_2^-
25	C_2H^-
32	S^-
33	HS^-
36	C_3^-
37	C_3H^-
38	$C_3H_2^-$
41	C_2HO^-
44	CS^-
45	CHO_2^-, CHS^-
48	C_4^-, SO^-
49	C_4H^-
51	$C_4H_3^-$
56	C_2S^-
57	C_2HS^-
61	C_5H^-
62	$C_5H_2^-$
64	SO_2^-

73	C_6H^-
75	$C_6H_3^-$
80	SO_3^-
81	C_4HS^-
83	$C_4H_3S^-$
92	C_5S^-
93	C_5HS^-
97	C_8H^-
105	C_6HS^-
129	C_8HS^-

Table S2. Selected peaks and their assignments observed in positive-ion ToF-SIMS spectra of the PTh films.

Positive ion mass spectrum *m/z*	Possible ion fragment/Possible structure
1	H^+
12	C^+
15	CH_3^+
27	$C_2H_3^+$
29	$C_2H_5^+$
39	$C_3H_3^+$
41	$C_3H_5^+$
43	$C_3H_7^+$
51	$C_4H_3^+$
55	$C_4H_7^+$
57	$C_4H_9^+$
67	$C_5H_7^+$
69	$C_5H_9^+$
77	$C_6H_5^+$
81	$C_6H_9^+$
83	$C_5H_7O^+$
85	$C_4H_5O_2^+$
91	$C_7H_7^+$

93	$C_7H_9^+$
95	$C_7H_{11}^+$
107	$C_7H_7O^+$

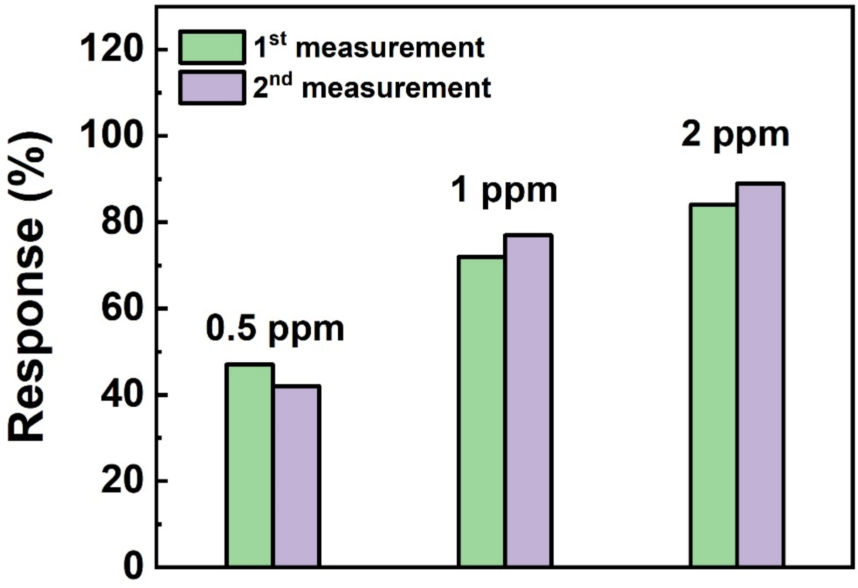

Figure S4. Repeatability of the sensors based on I_2-doped PTh films at different NO_2 concentrations.
